# Diversification versus Specialization in Complex Ecosystems

**DOI:** 10.1371/journal.pone.0112525

**Published:** 2014-11-10

**Authors:** Riccardo Di Clemente, Guido L. Chiarotti, Matthieu Cristelli, Andrea Tacchella, Luciano Pietronero

**Affiliations:** 1 IMT Institute for Advanced Studies Lucca, Lucca, Italy; 2 Istituto dei Sistemi complessi ISC-CNR, UOS Sapienza, Roma, Italy; 3 Dipartimento di Fisica, Università di Roma “Sapienza”, Roma, Italy; 4 London Institute for Mathematical Sciences, London, United Kingdom; Field Museum of Natural History, United States of America

## Abstract

By analyzing the distribution of revenues across the production sectors of quoted firms we suggest a novel dimension that drives the firms diversification process at country level. Data show a non trivial macro regional clustering of the diversification process, which underlines the relevance of geopolitical environments in determining the microscopic dynamics of economic entities. These findings demonstrate the possibility of singling out in complex ecosystems those micro-features that emerge at macro-levels, which could be of particular relevance for decision-makers in selecting the appropriate parameters to be acted upon in order to achieve desirable results. The understanding of this micro-macro information exchange is further deepened through the introduction of a simplified dynamic model.

## Introduction

Countries and firms are fundamental actors sharing complex economic and social ecosystems. Their evolutive paths lead to structurally different scenarios: firms are specialized entities while countries, as recently shown, are diversified [1], [2]. This raises a question on the mechanisms driving specialized entities to organize themselves into diversified super-structures. Is diversification a matter of size, of time horizon, or both? Are there other hidden dimensions governing the diversification process?

A similar scenario holds in biological ecosystems [3]: species (firms) tend to be substantially specialized, while groups of species competing on the same ecosystem (countries), appear to be diversified. Inspired by this argument in this paper we investigate the key mechanisms this picture is grounded on. It has been recently shown that this kind of analogy between economic and biological systems could gives rise to fruitful insights on elementary mechanisms [4].

Identifying the diversification drivers at the various scales is a challenging task in all disciplines since diversification processes are ubiquitous in nature [5] and economic systems [6], [7]. In our view economic ecosystems represent an ideal (paradigmatic) playground for an empirical investigation.

We therefore analyze the distribution of revenues across production sectors of quoted firms aggregated by country (Bloomberg database [8], [9]). Not surprisingly the analysis confirms that country competitiveness is mainly driven by diversification of productive systems, while firms' competitiveness is mainly a matter of specialization. The macroscopic signature of these macro-micro level discrepancies is reflected by the nested triangular structure of the country-sector binary matrix contrasting the essential randomness of the firm-sector binary matrix (see Methods section).

We argue that this is a specific observation of a general feature of complex systems: the shift from the macro to the micro level generally entails the loss of those features characterizing the former level. As in biology [10], the emerging diversification at macro level cannot be properly addressed at the level of individual species/firms. However, the environment in which the micro level is embedded preserves a sort of a macro level *memory* which enables to identify those micro level features that could emerge at larger scales [11].

**Figure 1:**
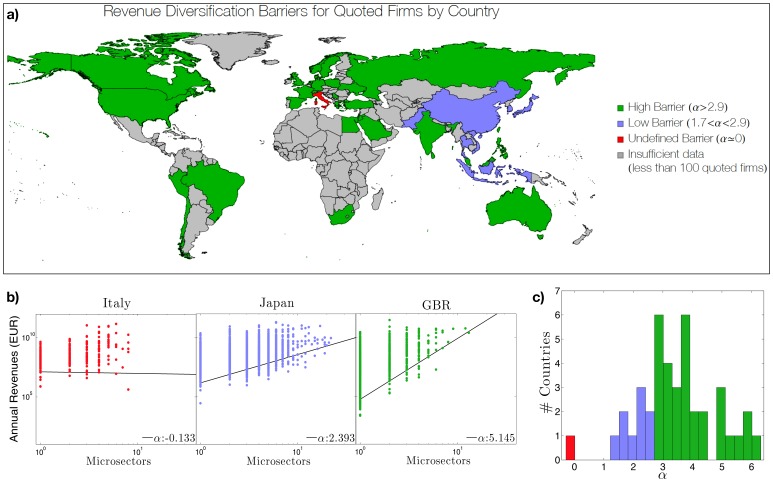
Revenue diversification barrier *α*. **a.** The worldwide distribution of the *revenue diversification barrier α*. The *α* tends to reflect geographical proximity and to cluster at the macro regional level. **b.** The scatter plot of firm revenues against firm diversification for thee paradigmatic countries. Except for Italy, the data draw a peculiar shape with a clear lower boundary. The angular coefficient of this linear boundary is what we define as the *revenue diversification barrier α*. **c.** The histogram of *α*. Colors are consistent with those used in panels a. and b.

Guided by this idea we show that, in the specific case of economic ecosystems, the microscopic feature emerging at the macro scale is the firm's diversification barrier *α* (see fig. 1). Moreover the *α*'s of different countries aggregate on macro-regional (multi-country) scale. This *zoom-in zoom-out* framework thus enables the identification of the proper micro-variable selecting the emerging (aggregated) macro-properties. This is of particular relevance in socio-economic systems, since it may help decision-makers to select the correct variable to be acted upon at the (micro) specialized level, in order to achieve desirable results at the (macro) diversified level.

In this respect the traditional economic literature has extensively studied the effect of institutions, policies and economic environments under which diversification has an impact on firm revenues [12]–[14]. However, the general picture which emerges from the standard approach is usually non conclusive as to whether diversification patterns affect firm revenues. Instead as mentioned, in the present work, we find that firm revenues are correlated to diversification, but the signature of this correlation appears in a highly non-trivial way as a selection rule which prevents firms from occupying a part of the diversification-revenues plane. We argue that the subtleness of this dependence - namely that high diversification implies high revenues while high revenues does not imply diversification. - is at the basis of the strongly debated economic literature about this field. We explored possible correlation between firms diversification and their size as measure by the number of employes without finding any significant signal.

We also propose a simple mathematical model mimicking the firms diversification dynamics in which firms evolve via a random walk in a random potential. Firm's survival rate depends on the values of the potential in the state reached by a particular firm environment in which firms compete. Surviving firms tend to diversify in time with a given probability. Such a minimal model is able to reproduce the main features observed in the data analysis.

## Results

The dataset we use consists of annual revenues of quoted firms disaggregated into Bloomberg's sector code and downloaded in May 2013. The database contains about 38000 firms and about 2000 sectors.

We proceed similarly to the work of [2] where an archival export dataset is considered to measure intangible assets determining the competitiveness of countries. It is worth noticing that in both analyses the datasets were not collected with the purpose of the analyses in which they were subsequently used.

As previously mentioned, the identification of the diversification drivers at the various scales is a challenging task in all disciplines. In Economics, in particular, it is unclear, but crucial, how the dynamics at micro-level determines the one at the macro-level and *vice versa*. This paper aims to shed some light on this very relevant question which affects how the economy should support the concrete implementation of economic policy decisions with a more scientific grounding.

The analysis confirms the recent finding [2] that country competitiveness is mainly driven by diversification of productive systems.

Coherently with the evidence of a triangular structure of country-product matrix in [1], [2], [15], [16], in the present analysis the same triangular feature is also found in the country-sector matrix obtained by aggregating firms on the basis of its legal address (see Information S1). The same matrix constructed at the firm level looses its nestedness and is similar to a random matrix with the same density (for further discussion see Methods section), reflecting firm specialization. This raises a rather fundamental question: what is the mechanism that organizes the information present into an almost random matrix, at the firm's level, in a nested matrix, at the country level?

To address this issue - within the general specialization trend for companies - we investigate whether there exist non trivial and country-dependent patterns of diversification. We identify in the *revenue diversification barrier* (hereafter *α*) the micro signature of these country-dependent patterns. It is interesting to note that this barrier *α* organizes itself at even higher level: this barrier tends to reflect geographical vicinity and to cluster at macro regional level. This can be observed in Fig. 1 panel a where we report worldwide distribution of *α*.

In panel b we report the scatter plot of firms' revenues (measured in EUR) against the firm diversification for three paradigmatic countries. With the exception of Italy, for all countries for which data are significant we observe a peculiar shape in which a clear lower boundary appears in the scatter plot. This means that while firms with high revenues can be either diversified or not, revenues of diversified firms are necessarily higher than non-diversified one. This suggests the existence of a *revenue diversification barrier* necessary to successfully diversify in a competitive market. In the double logarithmic space, the stiffness of this lower envelope naturally defines the barrier (for further details on the definition and robustness of the measure of *α* see Methods section).

In panel c we show the evidence for the nontrivial geographical clustering of the values of *α*. All the countries with low diversification barriers (blue) appear to belong to the Asian macro area with the notable exception of India, Hong-Kong and the Philippines. We speculate that these blue colored markets share a higher tolerance to diversification. In fact the diversification success of a firm is the result of the evolution in a competitive environment. The nature of this competition determines the stiffness of the barrier. On the other hand, the firms competing in green-colored markets are embedded in an environment which is operating a stronger selection of firms and consequently are characterized by a lower survival rate with respect to their diversification opportunities. Despite the fact that India, Hong-Kong and the Philippines are Asian countries, it is not surprising to find them among stiff markets because their value of *α* may reflect the strong anglo-saxon imprinting of the economic organization of these countries. Italy features an economy with different diversification dynamics. The substantially 0 value of *α* characterizing this market may mean that firm diversification is not driven by market selectiveness but rather by other exogenous mechanisms, which maybe related to an excess of family controlled companies [17] and/or incoherent companies aggregation [18] and/or protection mechanism reducing companies' failures [19].

**Figure 2:**
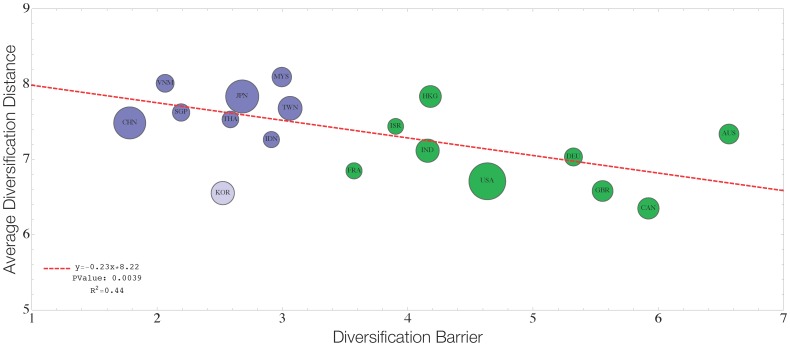
Diversification distance against revenue diversification barrier. The plot shows a clear negative correlation between these two variables. Blue and Green markets are clearly separated by both variables, suggesting that firms in diversification-prone markets tend to diversify more and more coherently (i.e. with a smaller diversification distance). South Korea (lighter blue) appears to be an outlier and removing it from the regression improves the quality of the fit (PValue decreases and *R* ^2^ increases).

To further characterize blue and green markets and consequently firm diversification patterns, we analyze the relation between *α* and the average diversification coherence of firms. The average diversification coherence is related to the typical distance among occupied sectors by a firm: the greater this typical distance, the lower the coherence (mathematical details of the definition of this measure are provided in Methods section). These two variables prove to be negative-correlated as shown in Fig. 2, indicating that the difference between blue and green markets is not only a matter of diversification barrier but also of diversification structure: firms operating in green markets tend to have revenues in sectors which are similar than those of firms living in blue markets. In terms of diversification, green markets are characterized by more coherent firms supporting the argument that selection rules are stricter in these economic systems.

### Model

We propose an extremely simplified model that embodies in our view the minimal traits necessary to shed light on the meaning of the revenue diversification barrier *α*. Firms are mimicked as random walkers moving in a random potential, seeking local minima. The height of such minima is representative of a firm's performance (due to its simplicity the model does not distinguish between firm performance and firm revenues): the lower the value of the potential, the better the performance. Markets (countries) differ in their tolerance (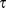) with respect to poor performances, i.e. in the probability for a firm to fail given its level of performance. Surviving firms, i.e. those with good performances, have the chance (*P_div_*) to diversify, while failed firms are replaced with new ones with the lowest possible level of diversification.

The random potential is a realization of a simple gaussian discrete random walk, with 0 mean and unit variance. We generate 100 equally spaced discrete points of the potential. The potential *V*(*x*) is then made periodic via a reflection, and is made continuous via a linear interpolation, the period being 200. Thus *V*(*x*) = *V*(*x*+k*200) holds for any real *x* and for any integer *k*. Finally *V*(*x*) is scaled to have maximum equal to 1 and minimum equal to 0.

Each firm starts at a random *x*_0_ coordinate and is made to evolve as a brownian particle in the potential defined by *V*(*x*). It seeks for local minima by evolving with the Metropolis-Hastings algorithm.

At each time step a proposal 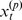 for a new value of *x_t_* is drawn from a gaussian distribution 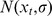. The parameter 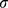 needs to be chosen such that the typical jump distance for a firm will be inside a typical local minima. This typical width is of order 1, by construction, thus we have chosen 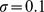. The proposal is then accepted with probability 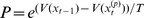. If the proposal is accepted we set 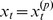 else *x_t_* = *x_t_*_-1_.

**Figure 3:**
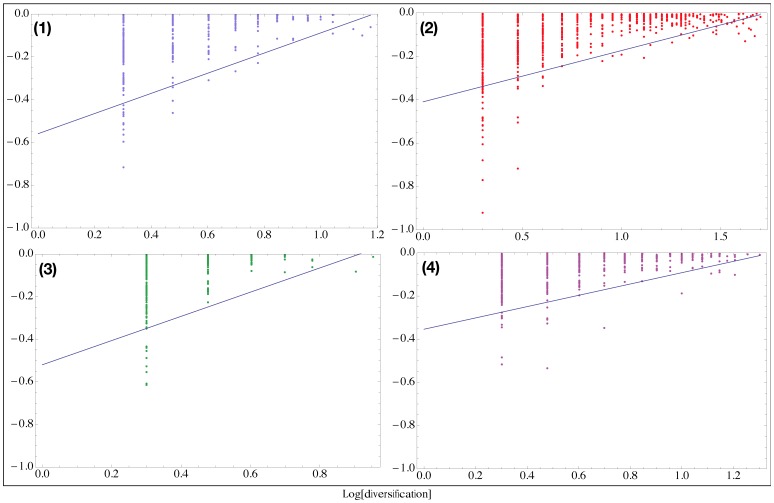
Performance versus diversification in the model. By making an analogy between the performance as defined in the present model and the revenues of a firm, it is possible to observe a lower boundary extremely similar to those observed in the real data, even in its functional form. The numbered labels indicate respectively the phase zone in Fig. 4.

**Figure 4:**
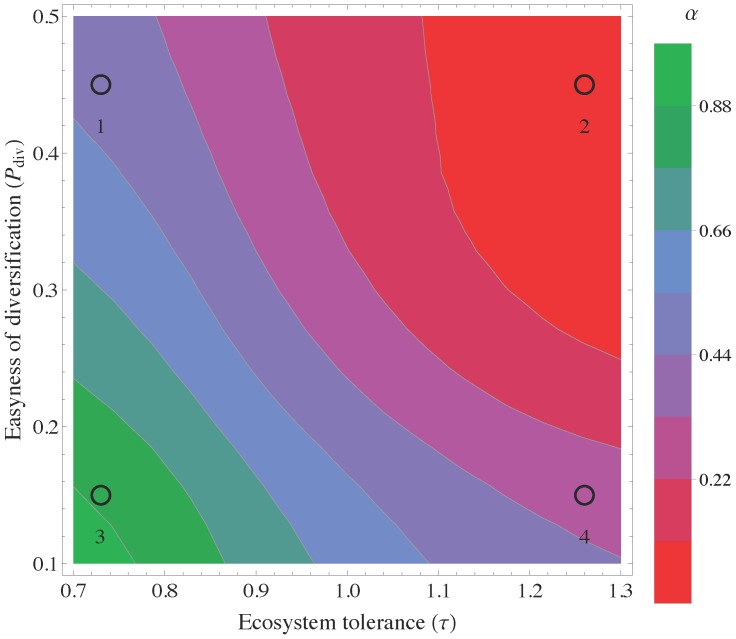
The phase diagram of the model obtained numerically. The diversification barrier *α* decreases in tolerant ecosystems and with increasing easiness of diversification *P_div_*. The numbers indicate the phase diagram zones explored by the model “countries” whose scatter plot of performance versus diversification are reported in Fig. 3. As green zone are populated by high diversification barrier “countries”, while purple zone by the lower barrier “countries”.

We define the performance of a firm as *P*(*t*) = 1−*V*(*x_t_*). Every 100 time-steps we compute the average performance 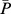 in such time window: the firm either survives with probability 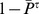 or fails. If the firm survives it has the chance to increase its diversification of 1, with probability *P_div_*. By making an analogy between the performance as defined in the present model and the revenues of a firm, we can observe in Fig. 3 how the model produces patterns very similar to those observed in the real dataset. Interestingly there is still a linear lower bound in the doubly logarithmic diversification vs. performance scatter plot. Within this model the diversification is clearly proportional to the life span of a given firm. The similarity between real data scatter plot and the model produced data can thus be interpreted in view of the question raised in the introduction: diversification is a dynamic process that develops over time and the boundary in the diversification-performance relation is set by the competitiveness of the environment in which the economic entities are immersed. In other words what we observe in real data is compatible with diversification being a dynamic process that goes on as long as a firm is able to survive. How long it will survive given its profits depends on the tolerance of the ecosystem. The differences in tolerance generates the differences in the diversification boundaries that we observe across countries. The values of *α* have a clear dependence on 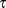 and *P_div_* as shown in the phase diagram in Fig. 4. In particular *α* decreases when the ecosystem tolerance increases. *P_div_* acts as a simple multiplier of the life span of a firm in determining its diversification.

## Discussion

The analysis of the distribution of firm revenues across production sectors aggregated by country manifests a peculiar triangular shape. This enables us to define a country dependent revenue diversification barrier “*α*”, which represents a novel macroscopic dimension driving the microscopic diversification process.

We have shown that this new macro feature shows a non trivial geographical clustering, which points out the importance and implication of the geo-political environment in the diversification patterns. *α* can be interpreted as the microscopic signature responsible for micro-macro information exchange showing that though the economic complexity methods it is possible to single out the microscopic variables governing the macroscopic dynamic.

Within our finding the microscopic firms' differentiation dynamics can be interpreted as a “*Darwinan*” competitive process in which the firms survival to diversification depends on the characteristics of the macroscopical (country like) environment. To further confirm this picture, a time dependent analysis on similar data is called for. Moreover, to better understand the meaning of this newly introduced dimension *α*, a comparison with other country dependent business environment indicators is called for and it will be implement in the future. These may include: Small and Medium Enterprises (SME) contributions to countries GDP, Global Competitiveness Index (GCI), and similar. We stres that the present analysis is restricted to quoted firms. It could be interesting to ask whether the influence of SMEs will affect the observed properties of *α*.

## Methods

### Triangularity vs. randomness

**Figure 5:**
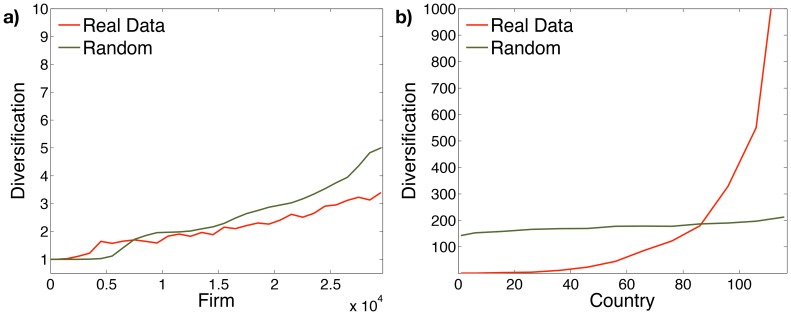
Comparison between the real data (red) and a random realization with same density (green). **a.** The firm-sector matrix exhibits a pattern similar to a random case emphasizing the firm' specialization. **b.** On the contrary aggregating the data on country level a non-random pattern emerges, corresponding to the presence of a nested structure.

The firm diversification level is the number of sectors developed by the firm. The real binary firm-sector matrix has a density close to 0.05. We generate a random matrix with same size and density of the real one. In figure 5a we show a comparison of the firm diversification, sorted by fitness [2], between the real data (depicted in red) and the random case (green). The two diversification trends show a similar pattern. This outlines the firms' high specialization and the absence of triangular structure in the matrix. Instead, in Fig. 5b, the real country-sector matrix, generated aggregating firms at country level on the basis of the legal address, exhibits a clearly nested (triangular) structure such as the country-product matrix [2].

### Definition of the revenue diversification barrier and its robustness

The diversification barrier *α* is measured as the slope of the lower boundary of the scatter plot of diversification vs. revenues in logarithmic space. The lower boundary is defined as the lower 5th percentile of the distribution of revenues for a given diversification level.

We check the sensitivity of *α* with respect to a variation of the percentile used to define the lower bound.

**Figure 6:**
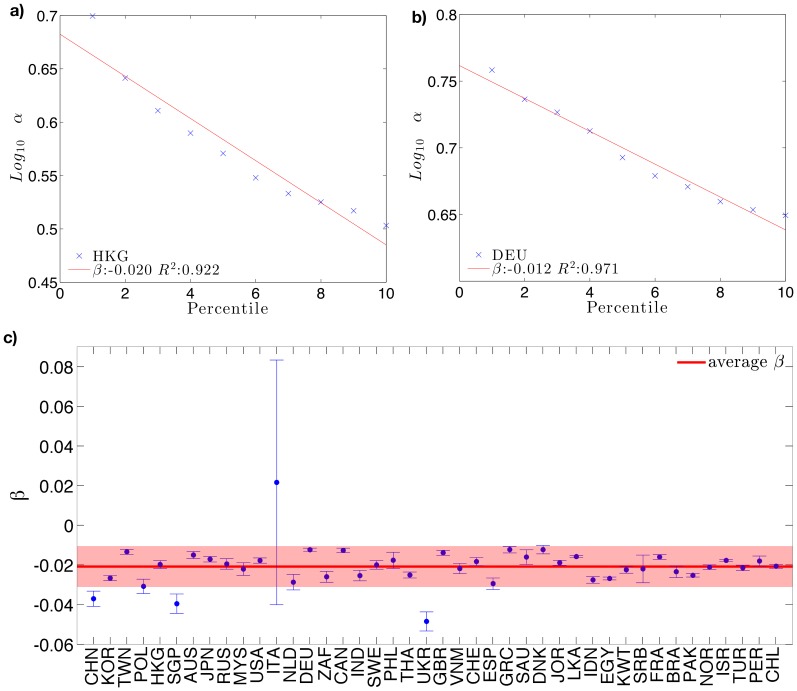
Dependency of *α* on different percentile cut-offs for two sample countries. **a–b.** The decay is well fitted by an exponential law *y* = *Ae^βx^* for all the countries examined. Values of *β* from regressions are shown in **c.** where each blue dot represents the coefficient *β* and its standard error for a specific country. The solid red line is the average value of *β* on all countries with more than 100 quoted firms. The shaded area in the plot marks one standard deviation. Most of the countries display a consistent decay of *α* with the percentile used thus making the particular choice of a percentile not relevant.

In fig. 6a–b different values of *α* for different percentiles are shown, for each country with at least 100 quoted firms. The plot clearly shows a decay trend which is common to (almost) all the countries. We then study in detail this decay of *α*. In figure 6c we show the angular coefficient (*β*) of a linear regression between the logarithm of *α* and the percentile, together with the respective standard error, for each country. For the majority of the countries *β* lies within one standard deviation from the average (red solid line). This shows that the consistency of our analysis is not affected by a particular choice of the percentile. Italy shows an anomalous sensitivity dependence with respect to other countries. The 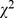 test over the *β* regressions in the fifth percentile accept the linear hypothesis at 95% for all the countries. The database we use it is available in Dataset S1.

### Diversification coherence

As mentioned, the BICS classification itself defines a topological distance between the codes, more precisely a tree. Each node in the tree corresponds to a more fine specification of the parent element.

Relying on this information we want to develop a measure of how coherently a firm is diversified. In particular we want to be able to weight diversification by a distance among the BICS categories in which diversification occurs: a company diversified in many very close subsectors might be considered less diversified than a company which has revenues only in two very distant sectors.

To this purpose we must take into account the fact that having revenues in a given sector and in one of its subsectors, at any level, does not add to the diversification. For this reason we cannot use the simple topological distance defined by the hierarchical tree implied by the BICS codes. Our approach is to define a new directed network, which is derived from the relations present in the BICS categorization, but with appropriate distances (or link weights). On such a network we use the total weight of the minimal (directed) spanning tree between all the nodes in which a company has revenues as a measure of its coherency.

To this end we need to define a distance (or link weights) that needs to have the following properties:

1. The distance between a sector and one of his subsectors must be 0 (producing pens and red pens does not add to diversification)
2. The distance between two subsectors of the same sector is proportional to the depth of the two subsectors (red pens and blue pens are more far apart than red pens with wooden body and red pens with plastic body)
3. As a consequence of the first property the distance between two sectors (A and B) and two of their respective subsectors (Aa and Bb) must be the same (pens are as distant from rulers as red pens are from metal rulers)
4. The distance between a subsector and its parent element sector must be infinite (to avoid 0 cost spanning trees between subsectors).

**Figure 7:**
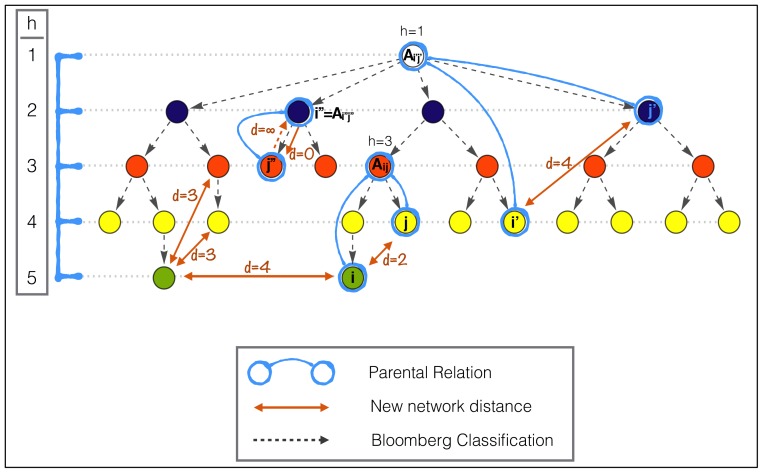
Examples of network distance as defined in Eq. 1.

**Figure 8:**
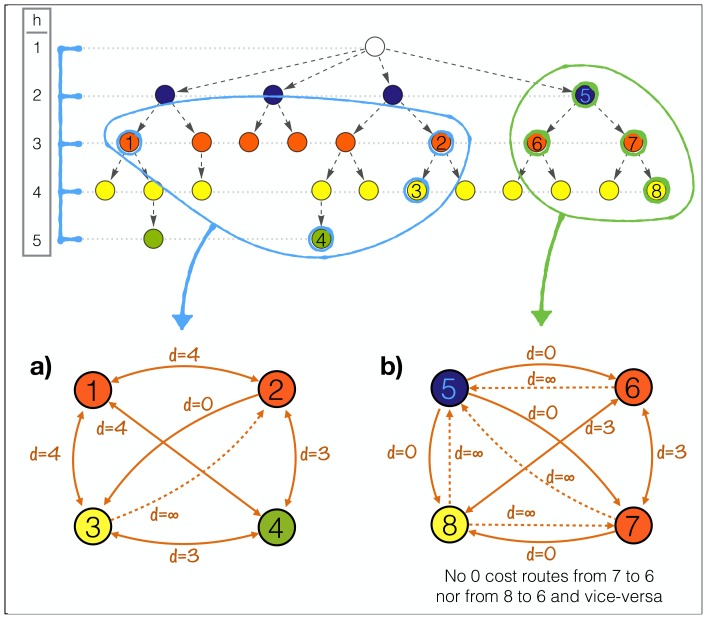
New resulting network. The resulting networks with link weights equal to *d_i,j_* for two hypothetical situations are shown in panels a and b. On these networks minimal spanning trees are determined via the Chu-Liu/Edmond's algorithm.

As depicted in Fig. 7 this translates in the fact that the distance between two nodes must be a function of depth of the nearest common parent element, except when one of the two nodes is a subsector of the other one, in which case the distance is asymmetric (0 or 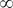). In formulae the distance is written as follows:
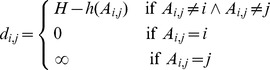
(1)where *A_i,j_* is the nearest common janitor to the nodes *i* and *j*, *h*(*A_i,j_*) is its depth in the tree and *H* is the total depth of the tree plus 1. The application of this definition is illustrated in Fig. 8 where the resulting networks, with link weights equal to *d_i,j_*, for two hypothetical situations are shown in panels a and b. On these networks minimal spanning trees are determined via the Chu-Liu/Edmond's algorithm [20]–[22].

## Supporting Information

Dataset S1 **The dataset.** The dataset to replicate the main findings of the article. In the first column there are the companies indicated simply by the country of domicile, in the second column the number of company micro sectors developed and in the third column its total amount of annual revenues in euros.
(ZIP)

Information S1 **Data specification.** The description of the dataset and how the data sanitation was performed.
(PDF)
