# Supplementary material for: Diversification versus Specialization in Complex Ecosystems
Source: PLoS One. 2014 Nov 10;9(11):e112525. doi: 10.1371/journal.pone.0112525 (PMC4226558; doi:10.1371/journal.pone.0112525)
Supplement: Information S1 — Data specification. The description of the dataset and how the data sanitation was performed. (PDF) [file pone.0112525.s002.pdf]

Supplementary Information

Diversification versus specialization in complex ecosystems

Riccardo Di Clemente[1,2]*, Guido L. Chiarotti[1], Matthieu Cristelli[1], Andrea Tacchella[1,3], & Luciano Pietronero[1,3,4]

[1] *Istituto dei Sistemi complessi ISC-CNR UOS Sapienza, Piazzale A. Moro 5, Roma Italy*
[2] *IMT Institute for Advanced Studies Lucca, Piazza S. Ponziano 6, Lucca, Italy*
[3] *Sapienza, Università di Roma, Piazzale A. Moro 5 Roma Italy.*
[4] *London Institute for Mathematical Sciences, 35a South St, Mayfair London United Kingdom.*

*Corresponding author: *E-mail address*: riccardo.diclemente@roma1.infn.it

S.1 Data specification

S.1.1 BICS hierarchical Classification system

The Bloomberg Industry Classification Systems (BICS) is a proprietary hierarchical classification system, which classifies firms' general business activities.

BICS for stock companies contains 10 *macro* sectors, which represent the broadest classification of general business activities. Each sector is further broken down into a hierarchical system of sectors (up to 8 levels of detail), which are classified into more narrowly defined business activities. The whole classification system counts up to 2294 unique sectors. Each *macro* sector is defined by a code composed by two digits. Sectors (or subsectors) are hierarchically defined by attaching further couples of digits to a parent element code. The deepest sector is defined by a 16-digits code. The figure S1 shows the first two levels of the Bloomberg BICS stocks hierarchical classification system for stock companies. The figure S2 shows the hierarchical tree system of BICS.

S.1.2 Dataset

The Bloomberg platform collects data about firms' revenues categorized using BICS. The data used in this work have been downloaded from Bloomberg platform on May 2013. The data contains information on annual revenues of 38274 traded firms broken down in 2294 sectors. The dataset covers 99 stock markets. Each firm is associated to its country of domicile, as reported by Bloomberg. the country of domicile is the country where the firm senior management is established legally. All the analyses have been performed on countries where at least 100 traded companies are domiciled. Those countries are listed in Table S1.

Level 1		Level 2	
**CODE**	**Macro Sector**	**CODE**	**First Level Microsector**
10	Communications	1010	Media Content
		1011	Telecom
11	Consumer Discretionary	1110	Apparel & Textile Products
		1111	Automotive
		1112	Consumer Discretionary Srvcs
		1113	Distributors
		1114	Home & Office Products
		1115	Leisure Products
		1116	Recreation Facilities & Srvcs
		1117	Retail Discretionary
		1118	Travel, Lodging & Dining
12	Consumer Staples	1210	Consumer Products
		1211	Dist/Whsl - Consumer Staples
		1212	Retail Staples
13	Energy	1310	Oil, Gas & Coal
		1311	Renewable Energy
14	Financials	1410	Asset Management
		1411	Banking
		1412	Institutional Financial Srvc
		1413	Insurance
		1414	Real Estate Oper & Srvcs
		1415	REIT
		1416	Specialty Finance
15	Health Care	1510	Biotech & Pharma
		1511	Health Care Facilities/Srvcs
		1512	Medical Equipment/Devices
16	Industrials	1610	Aerospace & Defense
		1611	Electrical Equipment
		1612	Engineering & Const Srvcs
		1613	Industrial Distribution
		1614	Machinery
		1615	Manufactured Goods
		1616	Transportation & Logistics
		1617	Transportation Equipment
		1618	Waste&Envrnmt Srvc Equip&Fac
17	Materials	1710	Chemicals
		1711	Construction Materials
		1712	Containers & Packaging
		1713	Forest & Paper Products
		1714	Iron & Steel
		1715	Metals & Mining
18	Technology	1810	Design, Mfg & Distribution
		1811	Hardware
		1812	Semiconductors
		1813	Software
		1814	Technology Services
19	Utilities	1910	Utilities

Figure S1: first two levels of the Bloomberg BICS stocks hierarchical Classification system for stock companies.

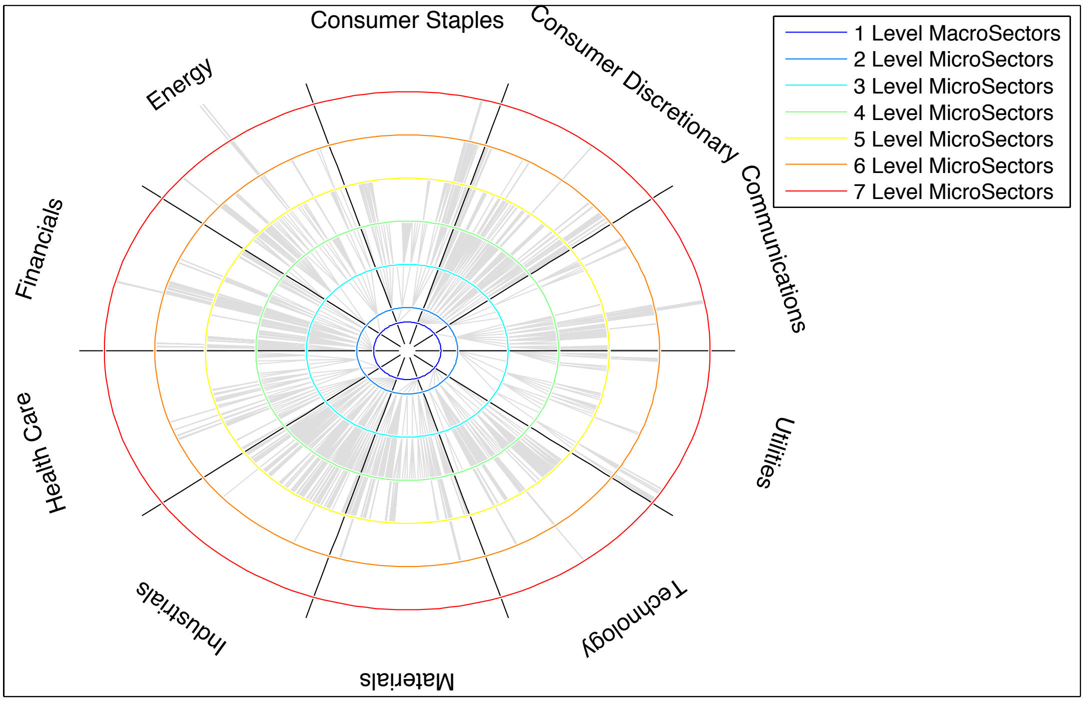

Figure S2: The Figure shows the reconstruction of BICS hierarchical tree system with its 2294 hierarchical Sectors. The colored lines show the classification levels.

S.1.2.1 Data sanitation

We organize our data in a firm-sector rectangular matrix. Most of the elements of this matrix are 0s. We perform various data sanitation procedures in order to extract relevant information from such matrix.

From a total of 216888 non-0 entries we eliminate 7333 negative revenues, since their meaning appears unclear. In particular we notice an abnormal number of repeated identical values, which clusterize on a national basis but span on different firms. The dataset provides for each subsector the aggregate of the firms' revenues on that particular subsector plus the sum of the revenues of the full hierarchy of subsectors below it. We perform the necessary subtractions to ensure that only

Country	# Companies	Country	#Companies
USA	4415	SWE	272
JPN	3366	POL	250
CHN	3112	ITA	245
TWB	1433	CHE	231
KOR	1341	GRC	213
IND	1338	LKA	210
HKG	1115	ZAF	198
CAN	1035	SRB	176
GBR	970	PHL	170
AUS	834	EGY	154
MYS	794	CHL	154
DEU	620	KWT	153
VNM	605	UKR	152
RUS	567	DNK	142
SGP	554	JOR	140
THA	483	NOR	135
IDN	439	ESP	122
FRA	437	NDL	118
ISR	406	PAK	115
TUR	297	PER	113
BRA	276	SAU	109

Table S1: Countries where at least 100 traded companies are domiciled.

the pertaining revenue is assigned to each subsector.
